# *Clerodendranthus spicatus* inhibits epithelial–mesenchymal transition of renal tubular cells through the NF-κB/Snail signalling pathway in hyperuricaemia nephropathy

**DOI:** 10.1080/13880209.2023.2243086

**Published:** 2023-08-21

**Authors:** Shouhai Wu, Meixia Yan, Junyi Liu, Yizhen Li, Ruimin Tian, Chuang Li, Lihuang Huang, Zhisheng Lu, Peng Xu, Wei Mao

**Affiliations:** a State Key Laboratory of Dampness Syndrome of Chinese Medicine, The Second Affiliated Hospital of Guangzhou University of Chinese Medicine, Guangzhou, China; b Department of Nephrology, Guangdong Provincial Hospital of Chinese Medicine, Guangzhou, China; c Guangdong Provincial Academy of Chinese Medical Sciences, Guangzhou, China; d The Second Clinical Medical College, Guangzhou University of Chinese Medicine, Guangzhou, China; e Guangdong Provincial Key Laboratory of Chinese Medicine for Prevention and Treatment of Refractory Chronic Diseases, Guangzhou, China

**Keywords:** Hyperuricaemic HK2, traditional Chinese medicine, lowering uric acid, renal protection

## Abstract

**Context:**

*Clerodendranthus spicatus* Thunb. (Labiatae) (CS), a perennial traditional Chinese medicinal herb that can reduce serum uric acid (sUA) levels and ameliorate renal function is widely used to treat hyperuricaemic nephropathy (HN).

**Objective:**

To investigate the molecular mechanism of action of CS in HN treatment using *in vivo* and *in vitro* experiments.

**Materials and methods:**

Sprague-Dawley rats were randomly divided into control, HN, CS and positive control allopurinol groups. The HN group was intraperitoneally injected with 750 mg/kg oxonic acid potassium (OA), whereas the CS group was injected with OA along with a gavage of CS (low dose 3.125 g/kg, high dose 6.25 g/kg) for five weeks. For *in vitro* studies, uric acid-treated HK2 cells were used to verify the therapeutic mechanism of CS in HN.

**Results:**

HN rats exhibit pathological phenotypes of elevated sUA levels and renal injury. CS significantly improved these symptoms and sUA (*p* < 0.05) and blood urea nitrogen (*p* < 0.01) levels, and dramatically improved renal tubular injury in HN rats. The IC_50_ value of UA (uric acid) in HK2 cells was 826.32 ± 3.55 μg/mL; however, 120 ng/mL CS had no significant cytotoxicity on HK2 cells. *In vivo* and *in vitro* studies showed that CS inhibited NF-κB phosphorylation and inhibited α-smooth muscle actin (α-SMA) and vimentin expression while increasing E-cadherin expression, suggesting that CS inhibited the fibrotic process in renal cells, thus protecting renal function.

**Discussion and conclusions:**

These findings provide a fundamental understanding of the application of CS in HN treatment to better guide clinical interventions.

## Introduction

Hyperuricaemia (HUA) is an abnormal metabolic syndrome caused by purine metabolism disorder (Huo et al. 2021), and the incidence of HUA has been continuously increasing in recent years (Dehlin et al. 2020). Previous studies have shown that both symptomatic and asymptomatic HUA can cause renal injury (Dissanayake et al. 2020). Urate reabsorption and excretion transporters play significant roles in HUA pathogenesis (Benn et al. 2018; Huo et al. 2021). Persistent HUA and urate deposition in the interstitial tissue of the renal medulla leads to chronic uric acid (UA) nephropathy, which activates the production of reactive oxygen species or stimulates the renin–angiotensin system (Yang et al. 2019), resulting in endothelial cell injury, high glomerular pressure, inflammatory reactions, renal tubular epithelial–mesenchymal transition (EMT) (Kang 2018) and eventually renal interstitial fibrosis (RIF) (Sheng and Zhuang 2020). RIF caused by HUA is known as hyperuricaemic nephropathy (HN). There are two pathways through which UA causes RIF. First, UA directly stimulates renal tubular cells (RTC) to secrete fibrogenic precursors and induces lysyl oxidase in renal tubular epithelial cells by inducing the nuclear factor-κB (NF-κB) pathway, which increases extracellular matrix synthesis, thus leading to interstitial fibrosis (Liu 2010). In the second pathway, UA aggravates existing fibrosis by upregulating α-smooth muscle actin (α-SMA) and type I collagen expression in the renal interstitial fibroblasts (Chen and Xu 2018).

*Clerodendranthus spicatus* Thunb. (Labiatae), a perennial herb, has been listed in the latest revision of ‘The Plant List’ (www.theplantlist.org) and Medicinal Plant Names Services (http://mpns.kew.org). CS, also known as *Orthosiphon aristatus* or *Kidney-Tea* in China, is a Dai traditional medicine from Yunnan Province, China. According to the Dictionary of Chinese Medicine, the slightly bitter and cool-natured CS can enter the liver, bladder and kidney (Luo et al. 2017). CS clears heat and dampness, removes urinary calculus and purifies water (Luo et al. 2018). Because of these features, CS can be used for the prevention and treatment of acute and chronic nephritis, cystitis, urinary calculi and gout. In Southeast Asia, CS is widely used to treat tonsillitis, rheumatism, diabetes, gonorrhoea, epilepsy, hypertension, menstrual disorders, syphilis, kidney stones, gallstones, oedema, rash, fever, influenza, hepatitis and jaundice (Zhou et al. 2017; Luo et al. 2018). In Europe and Japan, CS has been used as a health-promoting tea (Zhou et al. 2017). CS contains terpenoids, flavonoids and phenolic acids (Zheng et al. 2012). Current research indicates that CS possesses diverse bioactive properties, including antioxidant (Cai et al. 2018), anti-obesity (Seyedan et al. 2017), anti-inflammatory (Tabana et al. 2016), diuretic (Arafat et al. 2008), serum uric acid (sUA)-reducing (Chen et al. 2020) and nephroprotective (Xu et al. 2020) activity. Moreover, studies have highlighted that diterpenoids from CS reduce rat renal fibrosis via the transforming growth factor-β pathway (Luo et al. 2018). CS also has a strong anti-inflammatory effect on the lipopolysaccharide-induced inflammatory response in renal epithelial cells (Luo et al. 2018). However, the mechanism of action of CS in HN therapy remains unclear.

EMT is considered an early marker of renal fibrosis (Sun et al. 2016), and can be targeted because of its reversible nature (Kang 2018). During EMT, RTC lose their epithelial phenotype and undergo phenotypic transformation into mesenchymal cells (Zha et al. 2019; Guo and Zhang 2020). This process usually involves the downregulation of E-cadherin and cytokeratin expression and the upregulation of α-SMA, β-catenin and vimentin expression (Turini et al. 2019). However, the role of CS in the EMT in HN remains unclear.

NF-κB is composed of transcription factor families and plays a key role in immunity, inflammation (Sun 2017), cell proliferation and differentiation (Zhou et al. 2020). It has been found that NF-κB not only induces an inflammatory reaction of the kidney but also mediates EMT through various pathways (He et al. 2019). It can also mediate the transcription and stabilization of the Snail protein, leading to inflammation or EMT (Zhang et al. 2018). Snail was first identified as a transcription factor in *Drosophila melanogaster*; it regulates E-cadherin and is closely associated with EMT (Serrano-Gomez et al. 2016). Snail protein can downregulate epithelial markers, such as cytokeratin, and upregulate mesenchymal markers, such as vimentin and fibronectin (Turini et al. 2019). Thus, although NF-κB is involved in EMT, it is also not entirely clear whether CS participates in EMT in HN via activation of the NF-κB pathway.

In this study, we sought to understand the therapeutic role of CS in the EMT environment of HN through the NF-κB/Snail pathway and to investigate its molecular mechanism.

## Materials and methods

### Animals and HN model

Adult male Sprague-Dawley (SD) rats (200 ± 20 g) were obtained from the Medical Animal Experimental Center of Guangdong Province (Guangzhou, China). The rats in the HN model were administered 750 mg/kg oxonic acid potassium (OA) (2207-75-2, Sigma, St. Louis, MO) and 300 mg/kg UA (U2625, Sigma, St. Louis, MO), dissolved in 0.3% carboxymethyl cellulose (CMC-Na, C299502, Aladdin, Shanghai, China) solution via a gavage. Upon continuous administration for five weeks, the rats showed a hyperuricaemic renal injury phenotype, termed the HN model. All animal experiments were approved by the Animal Review Board of the Guangdong Provincial Hospital of Chinese Medicine (approval number: 2020068).

### Preparation of *Clerodendranthus spicatus*

CS was purchased from Kangmei Pharmaceutical Company, Ltd. (Guangzhou, China). The water extract was prepared by immersing 1350 g of CS in 1350 mL of pure water for 30 min and then boiled for 75 min, filtrated to obtain the first extracts, then 1200 mL of pure water was added, and the mixture was boiled for another 45 min, filtrated to obtain the second extract. The two water extracts were evenly mixed, filtered, evaporated and concentrated to 1080 mL using a rotary evaporator (R220, SENCO, Shanghai, China). Finally, a 1.25 g/mL CS solution was obtained and frozen at −80 °C. For *in vitro* experiments, a hydroalcoholic extract of CS was used. Briefly, herbal pieces of CS (150 g) were decocted with pure water and concentrated to 150 mL to obtain a 1 g/mL water extract. Then, 420 mL of 95% ethanol was added to the water extract, and further filtered in a rotary steamer at 60 °C by vacuum filtration technology using ethanol, which had been evaporated to 150 mL, and placed in a freeze dryer for 48 h. Then, 1 g of the obtained freeze-dried powder of CS was diluted to 1 mg/mL in DMEM/F12 (Gibco, Carlsbad, CA, 11330) to obtain the CS hydroalcoholic extract; this was sterilized with a 0.2 μm filter screen, and stored at −20 °C in the refrigerator until further use. All drugs were stored at room temperature prior to use.

### Ultra-high-performance liquid chromatography (UHPLC) analysis

Standard compounds were dissolved in 50% methanol. Dry CS powder was diluted with ultrapure water to a concentration of 10 mg/mL. Chromatogram fingerprints were analysed using an UHPLC Acquity system (Waters, Milford, MA), with a photodiode array detector (scanning from 200 to 400 nm) recording at 323 nm. The UHPLC conditions included a column ACQUITY BEH C18, 100 × 2.1 mm, 1.8 μm particle size (Waters, Milford, MA); column temperature, 35 °C; injection volume, 5 μL; mobile phase (A) 0.1% formic acid (B) acetonitrile; flow rate, 200 μL/min; gradient B, 5% (0 min), 5% (5 min), 20% (15 min), 28% (25 min), 60% B (30 min), 5% (32 min) and 5% (33 min).

### Experimental grouping

The rats were divided into five groups (*n* = 10) and treated as follows: control group (Ctrl) treated with 0.3% CMC-Na; HN group treated with 750 mg/kg OA + 300 mg/kg UA; low-dose CS group (CS-L) treated with HN + 3.125 g/kg water extract of CS; high-dose CS group (CS-H) treated with HN + 6.25 g/kg water extract of CS; and positive control group (AP) treated with HN + 5 mg/mL allopurinol. The drugs were administered continuously for five weeks. The dosage of CS was calculated according to the ‘conversion coefficient of dose per kilogram body weight of animals and humans.’ The dosage of the CS-L group was equivalent to a human dose of 30 g/d, and that of the CS-H group was equivalent to 60 g/d.

### Detection of blood biochemical indices

After the 5-week feeding, animals were euthanized and whole blood was harvested from the abdominal aorta 1 h after the last treatment. Serum was obtained after centrifugation, and assessed for sUA, serum creatinine (sCr) and blood urea nitrogen (BUN) levels. Simultaneously, kidneys were collected for histological examination and protein analysis.

### Cell viability assay

HK2 cells (Human Kidney-2, ATCC CRL-2190) were seeded in 96-well plates at a density of 70%. After induction with UA (0, 200, 400, 600, 800 and 1000 μg/mL) or CS (0, 15, 30, 45, 60, 75, 90, 105 and 120 ng/mL) for 48 h, 20 μL Cell Counting Kit-8 reagent (CCK8, Tongren Chemical Co., Ltd., Shanghai, Japan) was added to the cells for 2 h and incubated at 37 °C, 5% CO_2_. The optical density was measured at 450 nm using an enzyme gauge. Cell viability was calculated as follows: cell viability (%) = (treated/control) × 100.

### Luciferase and reporter assays

HK2 cells were incubated in a 24-well plate and treated with the specified plasmid and NF-κB-luciferase as the manufacturer’s instruction (IgK-IFN-luc, #14886, Addgene, Watertown, MA), together with pRL-TK (pRL-SV40P, #27163, Addgene, Watertown, MA). At 24 h post-transfection, luciferase activity was monitored using the Dual-Luciferase Reporter Assay (E1910, Promega, Madison, WI) according to the manufacturer’s instructions.

### Quantitative real-time polymerase chain reaction (qRT-PCR)

Total RNA was extracted using an RNeasy Plus Kit (Qiagen, Hilden, Germany), and cDNA was synthesized using a cDNA Reverse Transcription Kit (4368814, Thermo Fisher, Waltham, MA). Then, qRT-PCR was performed using the iTaq Universal SYBR Green (1725122, Bio-Rad, Hercules, CA). All data were normalized to β-actin expression levels. Primers used are listed in Table 1.

**Table 1.** Related to mRNA expression test, primer list for qRT-PCR.

Gene	Forward (5′–3′)	Reverse (5′–3′)
β-actin	CCTGGCACCCAGCACAAT	GGGCCGGACTCGTCATAC
Vimentin	TGAATGACCGCTTCGCCAACTAC	CTCCCGCATCTCCTCCTCGTAG
Snail	CGAGTGGTTCTTCTGCGCTA	GGGCTGCTGGAAGGTAAACT
Bcl-2	GCGACTCCTGATTCATTGGG	ACTTCCTCTGTGATGTTGTATTT
Bax	GGCCCTTTTGCTTCAGGGTT	CGGCGGCAATCATCCTCT

### Western blotting (WB) analysis

The cells were harvested in NP-40 (Thermo Fisher Scientific, Waltham, MA) with 1% protease inhibitors (Thermo Fisher Scientific, Waltham, MA) at 1 × 10^6^ cells/mL. The samples were sonicated and centrifuged at full speed for 15 min at 4 °C. Protein concentrations were calculated using the Pierce BCA Protein assay (Millipore #71285, Billerica, MA). Then, 4× loading buffer (Sigma-Aldrich, St. Louis, MO) was added to each sample and boiled at 100 °C for 5 min. Proteins were transferred to polyvinylidene fluoride (PVDF) membranes (Bio-Rad, Hercules, CA) and further incubated with the appropriate antibodies (p-NF-κB, CST, 3033s, Boston, MA; NF-κB, CST, 8242s, Boston, MA; Vimentin, Abcam, ab92547, Cambridge, UK; α-SMA, Abcam, 5694, Cambridge, UK; E-cadherin, Abcam, ab231303, Cambridge, UK; Snail, CST, 387, Boston, MA; β-actin, Millipore, SAB3500350, Billerica, MA), then combined with the relevant secondary antibody (anti-rat IgG, CST, #7077, Boston, MA; anti-human IgG, CST, # 32935, Boston, MA). Immobilon Western Chemiluminescent HRP Substrate (WBKLS, Millipore, Billerica, MA) was used for protein detection.

### Immunofluorescence (IF) staining

Cells were fixed with 4% paraformaldehyde for 1 h and blocked with a blocking buffer (Thermo Fisher #B10710, Waltham, MA) for 5 h at 4 °C. Cells were then stained with the primary antibody (E-cadherin, Abcam, ab231303, Cambridge, UK; Vimentin, Abcam, ab92547, Cambridge, UK; DAPI, Abcam, ab104139, Cambridge, UK) diluted according to manufacturer’s recommendation in blocking buffer, overnight at 4 °C. Cells were washed thrice with phosphate-buffered saline-tween (PBST) and stained with the secondary antibody (anti-mouse IgG, CST, #4408, Boston, MA; anti-rabbit IgG, CST, #4413, Boston, MA) for 2 h at 25 °C. The cells were washed with PBST to remove the secondary antibodies and then incubated in PBS. Images were obtained using a Zeiss LSM 710 Confocal Microscope (Oberkochen, Germany).

### Haematoxylin–eosin (H&E) and Masson staining

Rat kidney tissue was programmed for dehydration using a dehydration apparatus (Excelsior™ ES, Thermo Fisher, Waltham, MA), followed by paraffin embedding and sectioning (0.5 μm) and dewaxing using xylene for 10 min. For H&E staining, follow the steps in the product instructions of the haematoxylin and eosin staining kit (C0105M, Beyotime, Nantong, China) and observe and obtain pictures using the microscope (BX61, Olympus, Tokyo, Japan). For Masson staining, samples were processed according to the instructions of the Masson staining kit (BC-DL-002, Biochannel, Nanjing, China) and observed and obtained data using the same microscope.

### Flow cytometry

The cells were trypsinized and collected. After centrifugation at 1000 × *g* for 5 min, the supernatant was removed, and the cell pellets were washed once with PBS and resuspended in FACS buffer (Invitrogen #00-4222-26, Carlsbad, CA). For the apoptosis assay, flow cytometry was performed using a V-FITC Apoptosis Detection Kit according to the manufacturer’s instructions (Beyotime Annexin V-FITC Apoptosis Detection Kit, #C1062M, Nantong, China) on an Aria SORP cytometer (Becton Dickinson, Franklin Lakes, NJ) using CellQuest (Sebring, FL).

### Statistical analysis

Each experiment was independently replicated three times. Data are presented as the means ± standard deviation (SD). Statistical analysis was performed using SPSS20.0 (SPSS Inc., Chicago, IL) with one-way analysis of variance (Tukey’s test). Statistical significance was set at *p* < 0.05.

## Results

### Quality control and chemical profiles of CS

**Figure 1.**
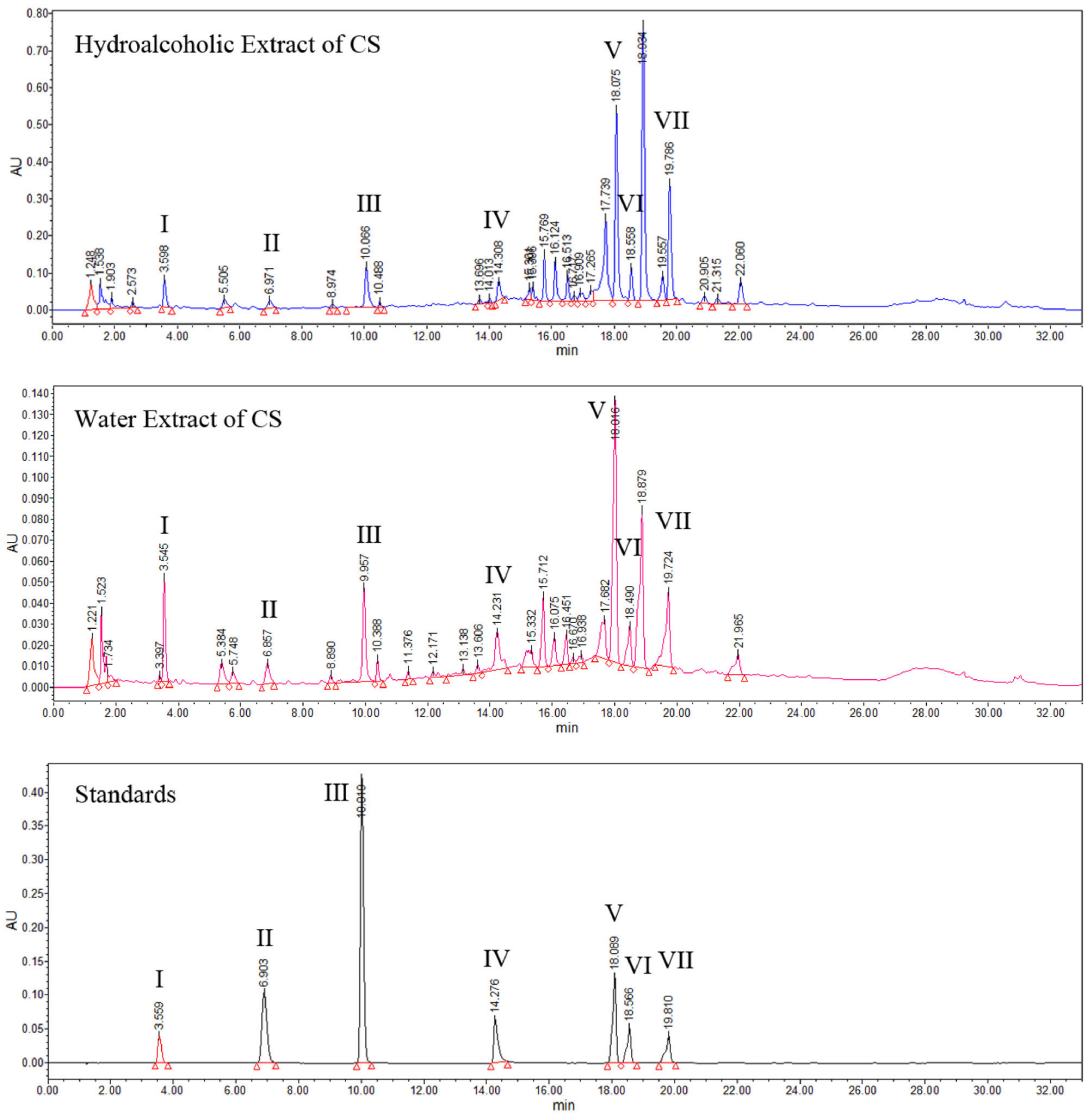
Identification of seven chemicals in *Clerodendranthus spicatus* using standard compounds. I: salvianic acid A, II: protocatechualdehyde, III: caffeic acid, IV: cichoric acid, V: rosmarinic acid, VI: lithospermic acid and VII: salvianolic acid B.

Compared with the standard compounds (Figure 1), seven chemicals, namely salvianic acid A, protocatechualdehyde, lithospermic acid, caffeic acid, cichoric acid, salvianolic acid B and rosmarinic acid, were identified in CS using UHPLC analysis (Table 2). These seven compounds were detected in both the water and ethanol extract; however, their concentrations were higher in the alcohol extract. Considering that water-extracted alcohol can also remove polysaccharides and other substances, the hydroalcoholic extract was selected for the cell experiments.

**Table 2.** Chemical profiles of *Clerodendranthus spicatus.*

Num.	Retention time (RT, min)	Maximum absorption wavelength (*λ*_max_, nm)	Compounds
I	3.55	279.2	Salvianic acid A
II	6.91	229.5	Protocatechualdehyde
III	10.0	322.2	Caffeic acid
IV	14.2	328.1	Cichoric acid
V	18.0	328.1	Rosmarinic acid
VI	18.5	253.1	Lithospermic acid
VIII	19.8	286.4	Salvianolic acid B

### CS relieved symptoms of HN in rats

**Figure 2.**
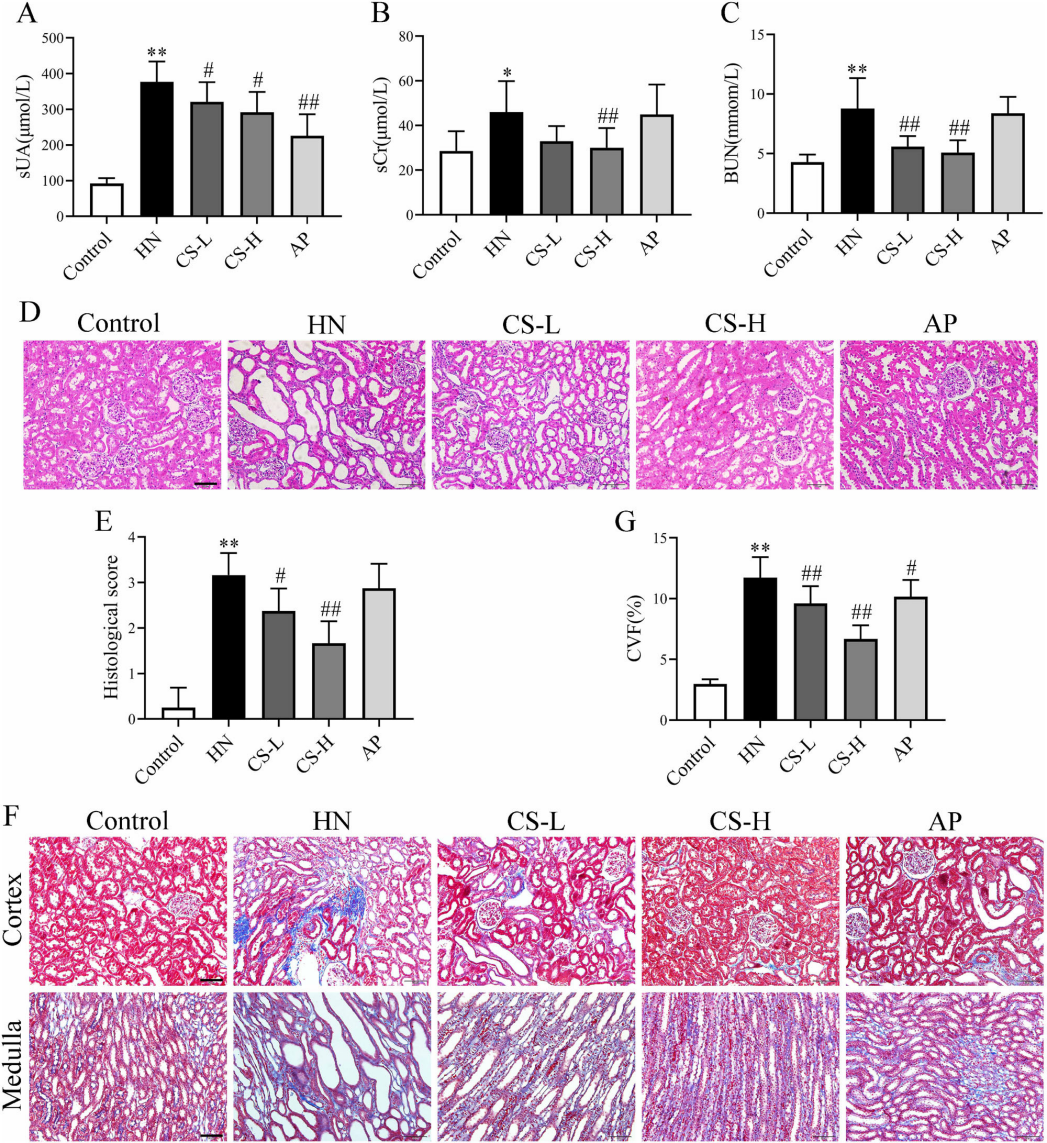
*Clerodendranthus spicatus* (CS) alleviated renal function parameters and histopathology in hyperuricaemic nephropathy (HN) rats. CS decreased the serum levels of serum creatinine (sCr) (A), blood urea nitrogen (BUN) (B), and serum uric acid (sUA) (C) in HN rats (*n* = 8). (D, E) Haematoxylin and eosin (H&E) staining showed that CS alleviated renal structural damage in HN rats. (F, G) Masson staining showed that CS improved renal damage and reduced the collagen volume fraction, with a significant decline in kidney score. Magnification: ×200. ^#^*p* < 0.05, ^##^*p* < 0.01 vs. HN group, *p < 0.05, **p < 0.01 vs. control group.

Levels of sCr, BUN and sUA are indicators of renal function. The sUA levels in HN rats were higher than those in the control group (*p <* 0.01; Figure 2(A)). The levels of sCr (*p <* 0.05, Figure 2(B)) and BUN (*p < 0.01*, Figure 2(C)) in the HN group were significantly higher than those in the control group, indicating that the HN rat model was successfully induced. The levels of sCr, BUN and sUA were decreased by CS, especially in the high-dose group (Figure 2(A–C)). Haematoxylin and eosin staining of the kidney tissue showed increased inflammatory cell infiltration, renal interstitial injury and renal tubule dilatation in the HN group compared to the control group. However, treatment with CS significantly increased and restored the renal pathology scores (Figure 2(D,E)). The degree of fibrosis was observably exaggerated, and collagen fibres were hypertrophic in HN rats compared to those in control rats (*p <* 0.01, Figure 2(F,G)). Notably, fewer collagen volume fractions were observed when rats were treated with CS (Figure 2(F,G)). Taken together, these results demonstrate that CS ameliorates renal injury in fibrotic rats. AP significantly reduced sUA levels (*p < 0.01*, Figure 2(A)) but exerted a protective effect on the kidney to a certain extent.

### CS inhibited EMT through NF-κB/Snail in HN rats

**Figure 3.**
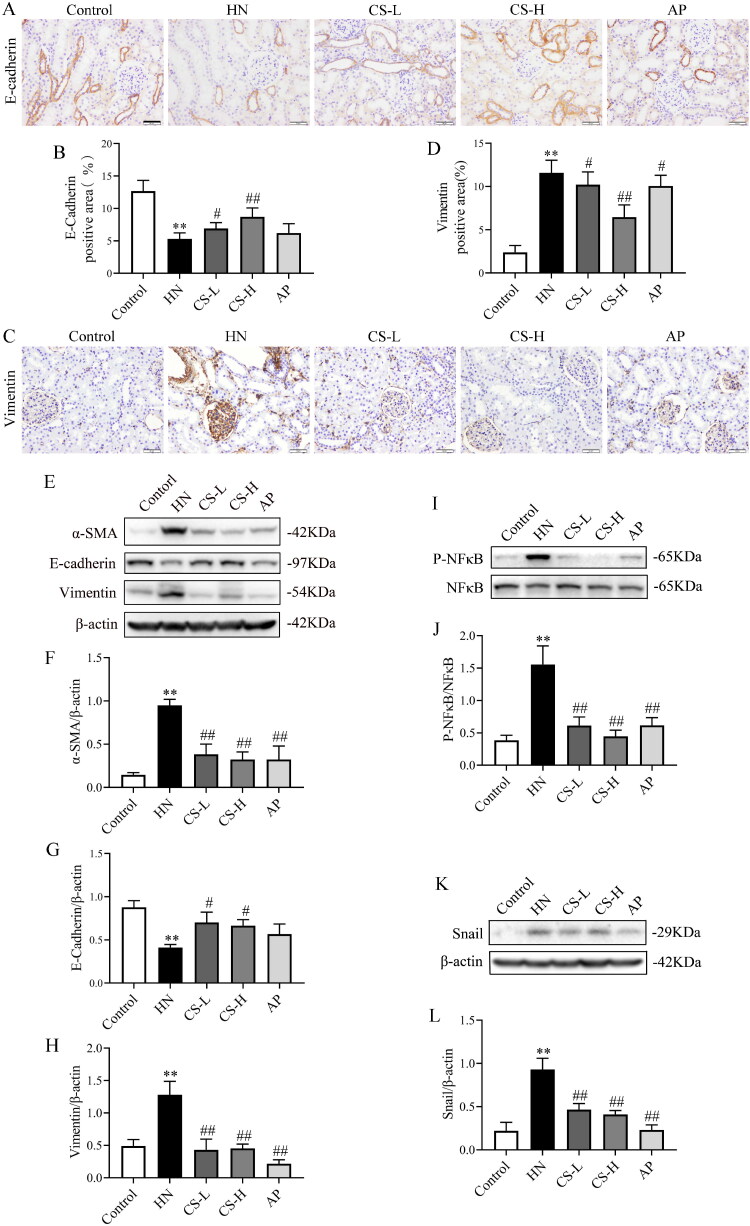
*Clerodendranthus spicatus* (CS) inhibited epithelial–mesenchymal transition (EMT) via the NF-κB and Snail pathways in hyperuricaemic nephropathy (HN) rats. Immunohistochemical analysis of E-cadherin (A, B) and vimentin (C, D) expression in HN rats (*n* = 3), magnification: ×200. Western blotting analysis of α-SMA (E, F), vimentin (E, G) and E-cadherin (E, H) expression in HN rats (*n* = 3). Western blotting was performed to determine the protein expression of p-NF-κB (I, J) and Snail (K, L) in HN rats (*n* = 3). ^#^*p* < 0.05, ^##^*p* < 0.01 vs. HN group, **p < 0.01 vs. control group.

Next, we assessed whether CS altered EMT and fibrosis. The expression of E-cadherin was significantly inhibited in HN rats (*p <* 0.01, Figure 3(A,B)), whereas CS significantly upregulated E-cadherin expression (Figure 3(A,B)). Vimentin levels were notably enhanced in HN rats compared to those in the control group, but was inhibited by CS treatment (*p <* 0.01, Figure 3(C,D)). In line with these findings, CS also downregulated α-SMA and vimentin and upregulated E-cadherin expression in HN rats (Figure 3(E–H)). These results show that CS inhibits EMT through NF-κB/Snail in HN rats.

### Effects of UA and CS on HK2 cells

**Figure 4.**
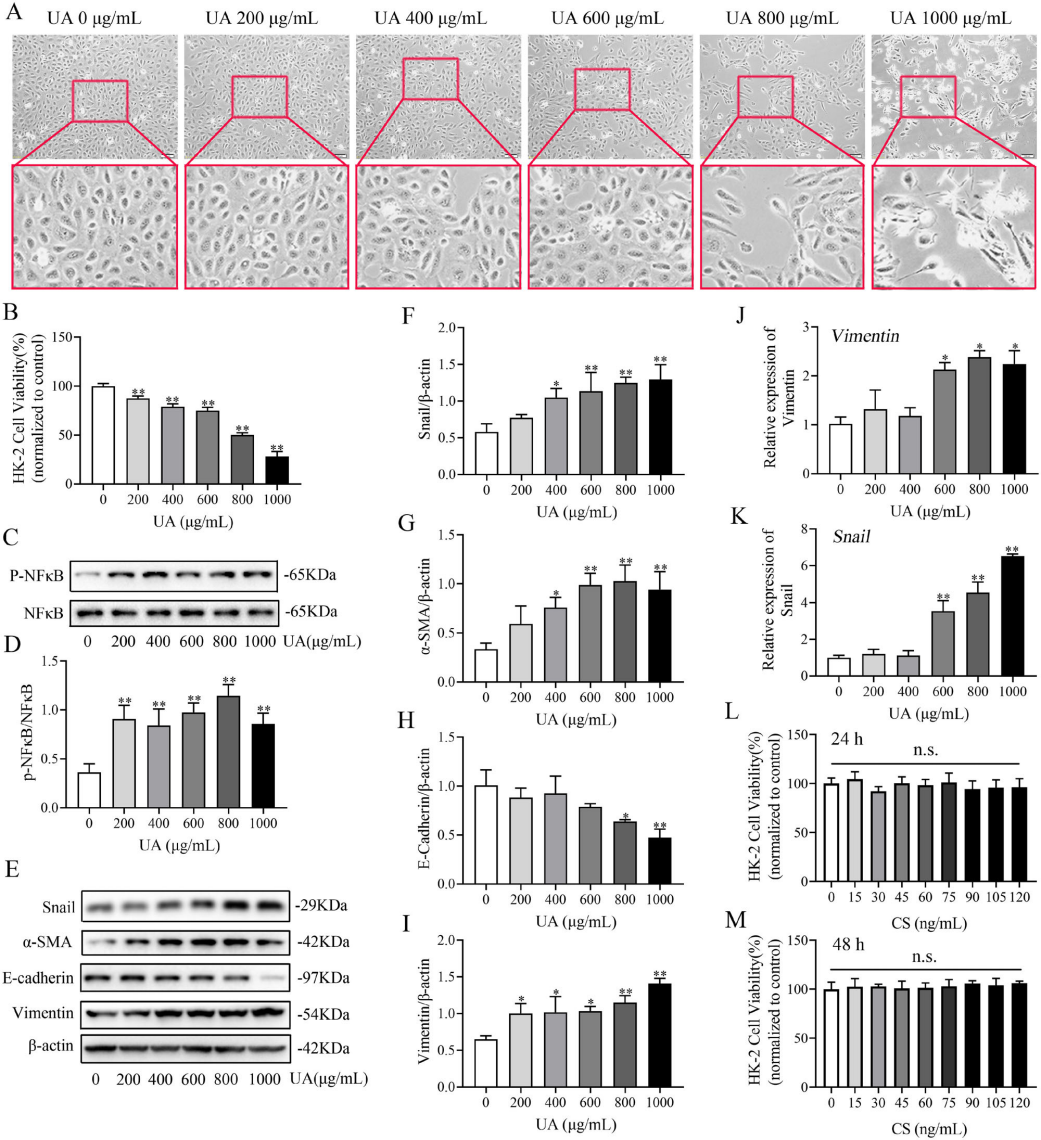
Effects of uric acid (UA) and *Clerodendranthus spicatus* (CS) in HK2 cells. The impact of UA on HK2 cell morphology. Magnification: ×200 (a). The viability rate of HK2 cells in UA (**p < 0.01 vs. control group) (B). The protein expression index of p-NF-κB (C, D) and Snail (E, F) examined by Western blotting (*n* = 3). *p < 0.05, **p < 0.01 vs. control group. Western blotting was adopted to monitor α-SMA (E, G), E-cadherin (E, H) and vimentin (E, I) expression and the band density (*n* = 3). The expression of *Vimentin* (J) and *Snail* (K) assessed by real-time qRT-PCR. The viability rate of HK2 cells incubated with CS at different concentrations for 24 h (L) and 48 h (M) (*n* = 3, n.s.: no significant).

HK2 cells were induced with different UA concentrations for 24 and 48 h, and the distance between the HK2 cells gradually widened in a dose-dependent manner. In particular, morphological changes in the 800 and 1000 μg/mL groups were obvious, with a characteristically long fusiform shape (Figure 4(A)). CCK8 assay was utilized to evaluate the effect of UA on HK2 viability, and the IC_50_ value of UA for HK2 was 826.32 ± 3.55 μg/mL (Figure 4(B)). UA significantly diminished HK2 cell viability, particularly at 800 μg/mL ant 48 h (*p < 0.01*; Figure 4(B)). The immunoblot data indicated that p-NF-κB (Figure 4(C,D)) expression drastically increased after 48 h of UA treatment. The p-NF-κB level reached its highest value on treatment with 800 μg/mL UA. Furthermore, UA increased the protein expression of Snail, vimentin and α-SMA in a concentration-dependent manner (Figure 4(E,F,G,I)), while E-cadherin expression was prominently decreased and was more pronounced at 800 μg/mL UA (*p < 0.05*, Figure 4(E,H)). qRT-PCR results revealed that *Vimentin* (Figure 4(J)) and *Snail* (Figure 4(K)) expressions were increased dose-dependently, especially in UA 600, 800 and 1000 μg/mL groups. These results suggest that UA induces EMT in HK2 cells. Treatment of HK2 cells with 800 μg/mL UA for 48 h was considered to induce EMT. The induced cells were labelled HUA-HK2 cells. We also analysed the effects of CS on HK2 cell viability. We found that the cell viability rate of each CS group was not significantly different at 24 h (*p > 0.05*, Figure 4(L)) or 48 h (*p > 0.05*, Figure 4(M)), confirming that HK2 cell viability was not affected by CS. Therefore, 100 ng/mL CS for 48 h was selected for subsequent experiments.

### CS alleviated HUA-HK2 EMT via the NF-κB/Snail signal pathway

**Figure 5.**
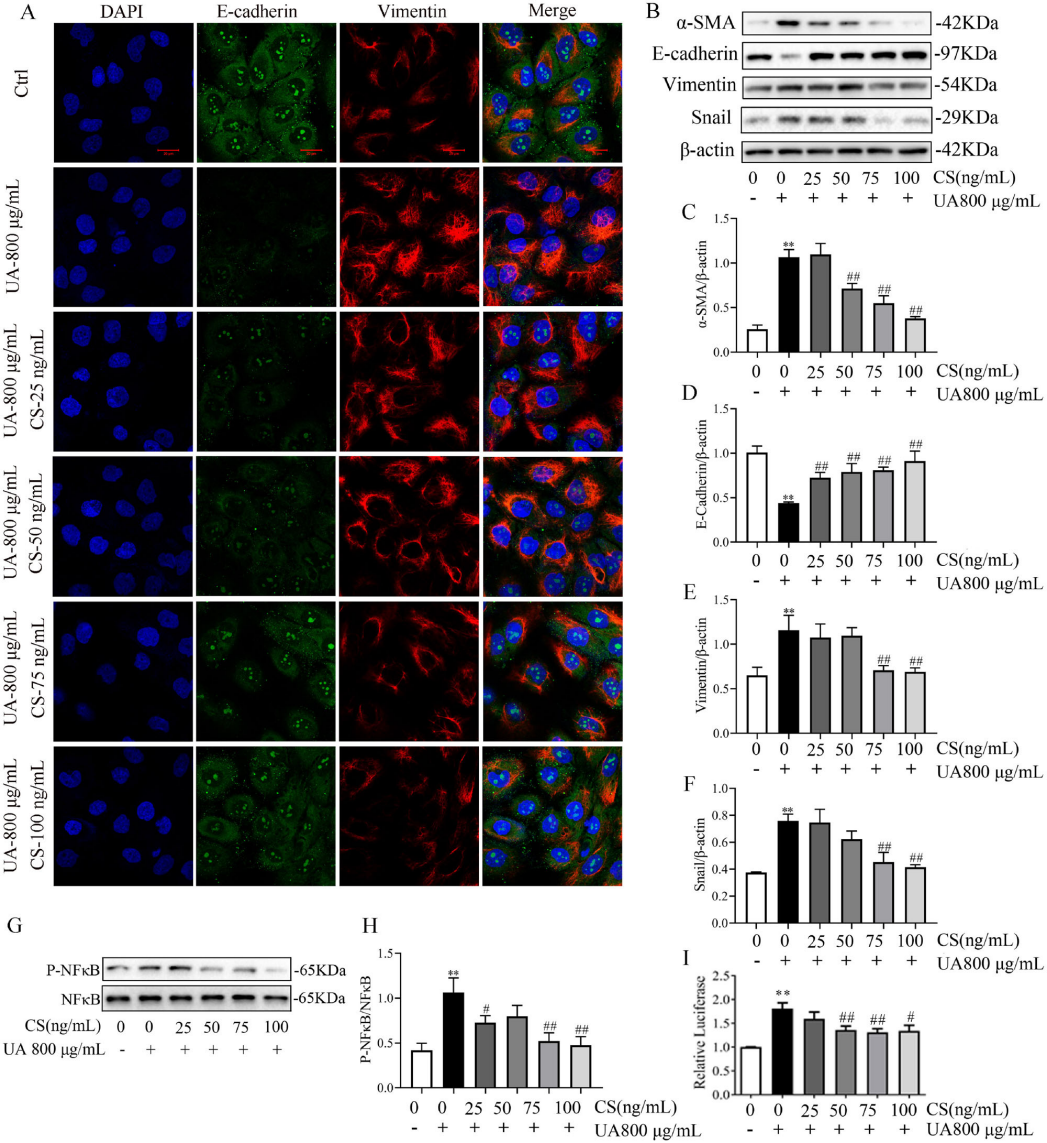
*Clerodendranthus spicatus* (CS) alleviated HUA-HK2 injury via the NF-κB/Snail signal pathway. (A) CS affects E-cadherin and vimentin expression in HUA-HK2 cells examined by immunofluorescence (*n* = 3). Magnification: ×200. Effects of CS on the levels of α-SMA (B, C), E-cadherin (B, D) and vimentin (B, E), and the band density (*n* = 3). Effect of CS on the protein expression index of p-NF-κB (G, H) and Snail (B, F) assessed by Western blotting (*n* = 3). ^#^*p* < 0.05, ^##^*p* < 0.01 vs. UA 800 μg/mL group, **p < 0.01 vs. control group. (I) Luciferase activity was tested using the corresponding kit.

Next, we investigated whether CS could alleviate HUA-HK2 injury. α-SMA and vimentin protein expression was dose-dependently inhibited by CS (Figure 5(B,C,E)), especially at 75 and 100 ng/mL (*p < 0.01*, Figure 5(C,E)). Meanwhile, relative to UA 800 μg/mL group, the attenuated E-cadherin expression was elevated by CS (Figure 5(B,D)). Furthermore, the protective effects of CS on HUA-HK2 injury were confirmed using IF (Figure 5(A)). IF data visualized that HUA-HK2 cells showed a significant decrease in E-cadherin expression and increase in vimentin expression compared to Ctrl cells and that CS could rescue these protein expression abnormalities. These results are consistent with those described above and with those of previous studies on HN rats. Besides, to explore the influence of CS on the NF-κB/Snail signalling pathway, WB was performed to evaluate NF-κB, p-NF-κB and Snail protein expressions. The results illustrated that CS intervention in HUA-HK2 cells increased p-NF-κB and Snail levels (Figure 5(B–F)). This proves that CS may inhibit HN progression via a mechanism involving the NF-κB and Snail signalling pathways. Employing the HK2/HUA-HK2 cells transfected with an NF-κB-luciferase reporter as a screening platform, we identified that UA could induce the expression of the NF-κB-dependent luciferase gene, while CS could significantly inhibit the expression of NF-κB (Figure 5(I)).

### CS reduces HUA-HK2 cell apoptosis

**Figure 6.**
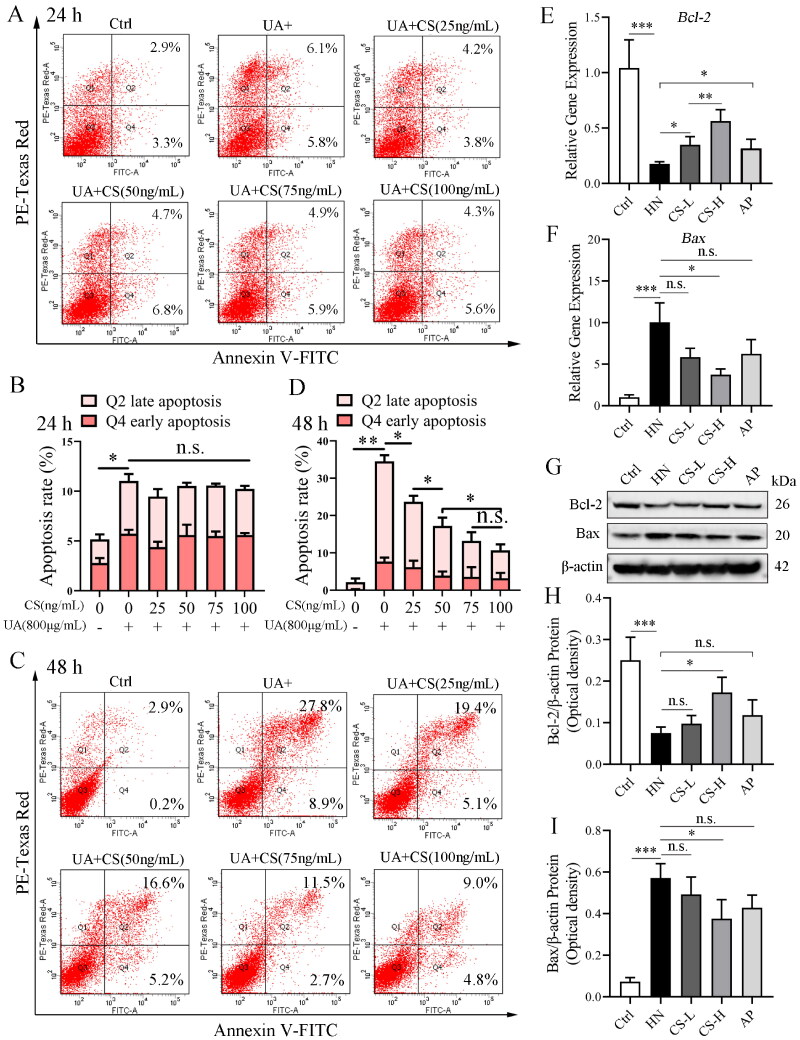
*Clerodendranthus spicatus* (CS) reduced HUA-HK2 cell apoptosis. (A) Apoptosis of HK2 cells in each experimental group at 24 h. (B) Statistical quantification of the percentage of apoptosis in each experimental group at 24 h (*n* = 3, *p < 0.05; n.s., no significant). (C) Flow cytometry detected the apoptosis at 48 h. (D) Statistical quantification of the percentage of apoptosis in each experimental group at 48 h (*n* = 3, *p < 0.05, **p < 0.01 n.s., no significant). (E, F) qPCR data of Bcl-2 and Bax mRNA expression in rat renal tubular cells of each experimental group (*n* = 3, *p < 0.05; **p < 0.01; ***p < 0.01; n.s., no significant). (G–I) Immunoblotting data of Bcl-2 and Bax protein expression in rat renal tubular cells of each experimental group (*n* = 3, *p < 0.05; ***p < 0.001; n.s., no significant).

High concentrations of UA induce apoptosis in HK2 cells. Apoptosis was observed when HK2 cells were cultured with 800 g/mL UA for 24 h (Figure 6(A)). Similarly, at 24 h, cells incubated with different CS concentrations also showed slight apoptosis; however, this was not significantly different from that observed in HUA-HK2 cells. The total percentage of apoptotic cells was approximately 12%, including 5.8% early apoptotic and 6.1% late apoptotic cells (Figure 6(A,B)). With an extended incubation time at high concentrations of UA, apoptosis of HK2 cells became crucial. At 48 h, the total percentage of apoptotic HUA-HK2 cells reached approximately 36%, including 8.9% early apoptotic and 27.8% late apoptotic cells (Figure 6(C,D)). Addition of CS significantly reduced apoptosis in HUA-HK2 cells; with an increase in CS concentration, the apoptosis rate decreased significantly (Figure 6(C,D)). These data indicate that CS protects RTC from high concentrations of UA. We also detected cell apoptosis in the medullary part of the rat kidney tissue, where the renal tubules are enriched, in all experimental groups. Furthermore, compared to that in the control group, the expression of Bcl-2 mRNA and protein in rat RTC in the HN group was significantly decreased, while the expression of Bax mRNA and protein was significantly increased (Figure 6(E–I)), suggesting apoptosis. Thus, CS treatment reduced apoptosis, especially when compared with the HN group in particular, the CS-L group demonstrated significantly reduced apoptosis (Figure 6(E–I)). We also observed that AP treatment moderately reduced apoptosis (Figure 6(E–I)).

## Discussion

According to the Great Dictionary of traditional Chinese medicine, CS has remarkable effects on clearing heat and dampness, diuresis and expelling renal stones. CS can reduce sUA levels, promote anti-inflammatory and diuretic effects, and protect the kidneys (Arafat et al. 2008; Xu et al. 2020). Research has shown that CS contains multiple ingredients such as caffeic acid derivatives, lipophilic flavones, flavonol glycosides (Wang et al. 2019), and compounds containing diterpenes, triterpenes and ursolic (Qi et al. 2017). Rosmarinic acid, one of the main components of CS, has been proved to have an inhibitory effect on xanthine oxidase (El-Desouky et al. 2019). In addition, metabolomic studies have confirmed that CS exerts protective effects against cisplatin-induced nephrotoxicity (Liu et al. 2020). Our current study verified that sUA levels in the HN group were 3–4 times higher than those in the control group, whereas sUA levels decreased significantly after intervention with CS decoction or AP. The sCr and BUN levels were effectively reduced in the CS group but not in the AP group, and the expression levels in the AP group were similar to those in the HN group. The findings from the pathological sections were consistent with the biochemical results. Compared to the HN group, the pathological manifestations in the CS group were alleviated, as shown by H&E staining. Masson staining showed that the collagen fibre volume was lower in the CS group than in the HN group, further confirming the protective effects of CS on the kidney.

Based on the EMT model, we verified the efficacy of CS. We chose HUA-HK2 cells induced with 800 μg/mL UA at 48 h as the condition to detect effects of CS on proliferation, EMT-related proteins and B/Snail pathway protein expression. The results showed that CS decoction had no cytotoxic effect on HUA-HK2 cells below 120 ng/mL. UA accelerates EMT in RTC by activating Snail and Slug and reducing E-cadherin expression (Ryu et al. 2013). Salidroside improves RIF by inhibiting the TLR4/NF-κB and MAPK signal pathways, implying that NF-κB and Snail can mediate the occurrence of renal EMT (Li et al. 2019). This is in accordance with our results, which showed that intervention with CS significantly upregulated cadherin expression and downregulated those of vimentin and α-SMA and the synthesis of P-NF-κB and Snail, thus reducing HUA-HK2 apoptosis. CS at 75 and 100 ng/mL had the most obvious inhibitory effects on EMT. Simultaneously, it was further confirmed that CS inhibited EMT progression in HUA-HK2 cells through the NF-κB/Snail pathway. However, in the present study, we did not screen or explore the effective chemical components of CS for HN therapy. We will further explore the effective chemical components of CS for the treatment of HN in future studies.

## Conclusions

CS inhibits the NF-κB/Snail signalling pathway in renal tissue and HUA-HK2 cells of rats with HN by decreasing EMT and apoptosis. In HN rats, CS reduced sUA levels and alleviated kidney damage, which is a potential mechanism of CS therapy for hyperuric acid nephropathy. Our findings suggest that CS has a renoprotective effect on HN and is especially suitable for patients with HUA, especially those with renal injury.

## Data Availability

Data are all contained within the paper.

## References

[CIT0001] Arafat OM, Tham SY, Sadikun A, Zhari I, Haughton PJ, Asmawi MZ. 2008. Studies on diuretic and hypouricemic effects of *Orthosiphon stamineus* methanol extracts in rats. J Ethnopharmacol. 118(3):354–360. doi: 10.1016/j.jep.2008.04.015. 18602231

[CIT0002] Benn CL, Dua P, Gurrell R, Loudon P, Pike A, Storer RI, Vangjeli C. 2018. Physiology of hyperuricemia and urate-lowering treatments. Front Med. 5:160–188. doi: 10.3389/fmed.2018.00160. PMC5990632 29904633

[CIT0003] Cai X, Xiao C, Xue H, Xiong H, Hang Y, Xu J, Lu Y. 2018. A comparative study of the antioxidant and intestinal protective effects of extracts from different parts of Java tea (*Orthosiphon stamineus*). Food Sci Nutr. 6(3):579–584. doi: 10.1002/fsn3.584. 29876108 PMC5980324

[CIT0004] Chen LL, Xu Y. 2018. Epigallocatechin gallate attenuates uric acid-induced injury in rat renal interstitial fibroblasts NRK-49F by up-regulation of miR-9. Eur Rev Med Pharmacol Sci. 22(21):7458–7469. doi: 10.26355/eurrev_201811_16287. 30468495

[CIT0005] Chen WD, Zhao YL, Sun WJ, He YJ, Liu YP, Jin Q, Yang XW, Luo XD. 2020. ‘Kidney Tea’ and its bioactive secondary metabolites for treatment of gout. J Agric Food Chem. 68(34):9131–9138. doi: 10.1021/acs.jafc.0c03848. 32786873

[CIT0006] Dehlin M, Jacobsson L, Roddy E. 2020. Global epidemiology of gout: prevalence, incidence, treatment patterns and risk factors. Nat Rev Rheumatol. 16(7):380–390. doi: 10.1038/s41584-020-0441-1. 32541923

[CIT0007] Dissanayake LV, Spires DR, Palygin O, Staruschenko A. 2020. Effects of uric acid dysregulation on the kidney. Am J Physiol Renal Physiol. 318(5):F1252–F1257. doi: 10.1152/ajprenal.00066.2020. 32223309 PMC7294331

[CIT0008] El-Desouky MA, Mahmoud MH, Riad BY, Taha YM. 2019. Nephroprotective effect of green tea, rosmarinic acid and rosemary on N-diethylnitrosamine initiated and ferric nitrilotriacetate promoted acute renal toxicity in Wistar rats. Interdiscip Toxicol. 12(2):98–110. doi: 10.2478/intox-2019-0012. 32206031 PMC7071836

[CIT0009] Guo N, Zhang J. 2020. Interleukin-17 promotes ovarian carcinoma SKOV3 cells via MTA1-induced epithelial-to-mesenchymal transition. Eur J Gynaecol Oncol. 41(1):70–74.

[CIT0010] He F, Fan MX, Jin YS, Wang HY, Ding L, Fan JF, Gu SS, Xu W. 2019. Sphingosine kinase 1 inhibition decreases the epithelial–mesenchymal transition and ameliorates renal fibrosis via modulating NF-κB signaling. Am J Transl Res. 11:5879–5887. 31632556 PMC6789211

[CIT0011] Huo S, Wang H, Yan M, Xu P, Song T, Li C, Tian R, Chen X, Bao K, Xie Y, et al. 2021. Urinary proteomic characteristics of hyperuricemia and their possible links with the occurrence of its concomitant diseases. ACS Omega. 6(14):9500–9508. doi: 10.1021/acsomega.0c06229. 33869930 PMC8047722

[CIT0012] Kang DH. 2018. Hyperuricemia and progression of chronic kidney disease: role of phenotype transition of renal tubular and endothelial cells. Contrib Nephrol. 192:48–55. 29393109 10.1159/000484278

[CIT0013] Li R, Guo Y, Zhang Y, Zhang X, Zhu L, Yan T. 2019. Salidroside ameliorates renal interstitial fibrosis by inhibiting the TLR4/NF-κB and MAPK signaling pathways. Int J Mol Sci. 20:1130. 30836660 10.3390/ijms20051103 PMC6429495

[CIT0014] Liu J, Wu Z, Han D, Wei C, Liang Y, Jiang T, Chen L, Sha M, Cao Y, Huang F, et al. 2020. Mesencephalic astrocyte-derived neurotrophic factor inhibits liver cancer through small ubiquitin-related modifier (SUMO)ylation-related suppression of NF-kappaB/Snail signaling pathway and epithelial–mesenchymal transition. Hepatology. 71(4):1262–1278. doi: 10.1002/hep.30917. 31469428 PMC7187412

[CIT0015] Liu Y. 2010. New insights into epithelial–mesenchymal transition in kidney fibrosis. J Am Soc Nephrol. 21(2):212–222. doi: 10.1681/ASN.2008121226. 20019167 PMC4554339

[CIT0016] Luo Y, Cheng LZ, Luo Q, Yan YM, Wang SM, Sun Q, Cheng YX. 2017. New ursane-type triterpenoids from *Clerodendranthus spicatus*. Fitoterapia. 119:69–74. doi: 10.1016/j.fitote.2017.04.001. 28392270

[CIT0017] Luo Y, Li XZ, Xiang B, Luo Q, Liu JW, Yan YM, Sun Q, Cheng YX. 2018. Cytotoxic and renoprotective diterpenoids from *Clerodendranthus spicatus*. Fitoterapia. 125:135–140. doi: 10.1016/j.fitote.2018.01.003. 29309828

[CIT0018] Qi J, Sun LQ, Qian SY, Yu BY. 2017. A novel multi-hyphenated analytical method to simultaneously determine xanthine oxidase inhibitors and superoxide anion scavengers in natural products. Anal Chim Acta. 984:124–133. doi: 10.1016/j.aca.2017.07.023. 28843555

[CIT0019] Ryu ES, Kim MJ, Shin HS, Jang YH, Choi HS, Jo I, Johnson RJ, Kang DH. 2013. Uric acid-induced phenotypic transition of renal tubular cells as a novel mechanism of chronic kidney disease. Am J Physiol Renal Physiol. 304(5):F471–F480. doi: 10.1152/ajprenal.00560.2012. 23283992

[CIT0020] Serrano-Gomez SJ, Maziveyi M, Alahari SK. 2016. Regulation of epithelial–mesenchymal transition through epigenetic and post-translational modifications. Mol Cancer. 15:18. doi: 10.1186/s12943-016-0502-x. 26905733 PMC4765192

[CIT0021] Seyedan A, Alshawsh MA, Alshagga MA, Mohamed Z. 2017. Antiobesity and lipid lowering effects of *Orthosiphon stamineus* in high-fat diet-induced obese mice. Planta Med. 83(8):684–692. doi: 10.1055/s-0042-121754. 27992939

[CIT0022] Sheng L, Zhuang S. 2020. New insights into the role and mechanism of partial epithelial–mesenchymal transition in kidney fibrosis. Front Physiol. 11:569322. doi: 10.3389/fphys.2020.569322. 33041867 PMC7522479

[CIT0023] Sun SC. 2017. The noncanonical NF-κB pathway in immunity and inflammation. Nat Rev Immunol. 17(9):545–558. doi: 10.1038/nri.2017.52. 28580957 PMC5753586

[CIT0024] Sun YB, Qu X, Caruana G, Li J. 2016. The origin of renal fibroblasts/myofibroblasts and the signals that trigger fibrosis. Differentiation. 92(3):102–107. doi: 10.1016/j.diff.2016.05.008. 27262400

[CIT0025] Tabana YM, Al-Suede FS, Ahamed MB, Dahham SS, Hassan LE, Khalilpour S, Taleb-Agha M, Sandai D, Majid AS, Majid AM. 2016. Cat’s whiskers (*Orthosiphon stamineus*) tea modulates arthritis pathogenesis via the angiogenesis and inflammatory cascade. BMC Complement Altern Med. 16(1):480. doi: 10.1186/s12906-016-1467-4. 27881135 PMC5122152

[CIT0026] Turini S, Bergandi L, Gazzano E, Prato M, Aldieri E. 2019. Epithelial to mesenchymal transition in human mesothelial cells exposed to asbestos fibers: role of TGF-beta as mediator of malignant mesothelioma development or metastasis via EMT event. Int J Mol Sci. 20(1):12. doi: 10.3390/ijms20010150. PMC6337211 30609805

[CIT0027] Wang S, Fang Y, Yu X, Guo L, Zhang X, Xia D. 2019. The flavonoid-rich fraction from rhizomes of *Smilax glabra* Roxb. ameliorates renal oxidative stress and inflammation in uric acid nephropathy rats through promoting uric acid excretion. Biomed Pharmacother. 111:162–168. doi: 10.1016/j.biopha.2018.12.050. 30579255

[CIT0028] Xu WH, Wang HT, Sun Y, Xue ZC, Liang ML, Su WK. 2020. Antihyperuricemic and nephroprotective effects of extracts from *Orthosiphon stamineus* in hyperuricemic mice. J Pharm Pharmacol. 72(4):551–560. doi: 10.1111/jphp.13222. 31910301

[CIT0029] Yang X, Gu J, Lv H, Li H, Cheng Y, Liu Y, Jiang Y. 2019. Uric acid induced inflammatory responses in endothelial cells via up-regulating(pro)renin receptor. Biomed Pharmacother. 109:1163–1170. doi: 10.1016/j.biopha.2018.10.129. 30551366

[CIT0030] Zha D, Wu S, Gao P, Wu X. 2019. Telmisartan attenuates uric acid-induced epithelial–mesenchymal transition in renal tubular cells. Biomed Res Int. 2019:3851718. doi: 10.1155/2019/3851718. 30993112 PMC6434300

[CIT0031] Zhang S, Huang Q, Cai X, Jiang S, Xu N, Zhou Q, Cao X, Hultström M, Tian J, Lai EY. 2018. Osthole ameliorates renal fibrosis in mice by suppressing fibroblast activation and epithelial–mesenchymal transition. Front Physiol. 9:1650. doi: 10.3389/fphys.2018.01650. 30524310 PMC6258720

[CIT0032] Zheng Q, Sun Z, Zhang X, Yuan J, Wu H, Yang J, Xu X. 2012. Clerodendranoic acid, a new phenolic acid from *Clerodendranthus spicatus*. Molecules. 17(11):13656–13661. doi: 10.3390/molecules171113656. 23165309 PMC6268606

[CIT0033] Zhou HC, Yang L, Guo RZ, Li J. 2017. Phenolic acid derivatives with neuroprotective effect from the aqueous extract of *Clerodendranthus spicatus*. J Asian Nat Prod Res. 19(10):974–980. doi: 10.1080/10286020.2016.1277707. 28140664

[CIT0034] Zhou Y, Cui C, Ma X, Luo W, Zheng SG, Qiu W. 2020. Nuclear factor kappa B (NF-kappaB)-mediated inflammation in multiple sclerosis. Front Immunol. 11:391. doi: 10.3389/fimmu.2020.00391. 32265906 PMC7105607

